# Annotations

**Published:** 1900-09-08

**Authors:** 


### ANNOTATIONS.

### Electric Trams and Suburban Industries.

In the recently issued Annual Report of the Chief Inspector of Factories there is a bit of information in regard to the effect of the tramway system in lessening the centralisation, and consequent overcrowding of both population and industries, which is of much importance. People are apt to regard the introduction of tramways, so far as it touches the question of overcrowding, merely as a means of enabling the city workers to live in the purer air of the suburbs far away from their work. We, however, have always maintained that if once cheap and rapid inter-communication were established, not only would the people be drawn to the outer ring, but that many of the industries would go there also, taking advantage of the cheaper labour to be found in the suburban districts. This is just what seems to be happening in Bristol. The factory inspector of that city says that, owing to the enterprise of the tramway company in extending their electric system of lines into the suburbs, a revolution is being rapidly effected in the domestic life of the workpeople. Several firms, such as manufacturers of tobacco, clothing, corsets, rope, &c., have already built fine modern factories on the outskirts of the town, and round these factories have sprung up rows of neat cottages, in some cases built and owned by the factory proprietors, who rent them to the workpeople. Here the people live in pure air instead of being cooped up in the narrow streets of the city, and yet, thanks to the tramways, they still have the advantages of city life, so far as regards shops, &c. Workers in the city also have the advantage of being able to sleep in the suburbs. Altogether, says Mr. Maitland, the inspector, few modern inventions have conferred greater benefits on the workpeople than have electric tramways, and if throughout the country, and especially in and around large cities, electric tramways receive the encouragement they deserve they may become an important factor in the solution of the overcrowding problem. These are our sentiments exactly. We do not limit, as some would, the benefits of the tramways to the mere facilities they would offer to the city worker in the way of having his home in the suburbs. The far greater benefit which will result from the extension of the tramways will be that the towns themselves will spread out. With the increased facilities of intercommunication between one part and another, the tendency of the industries as well as of people to crowd into the centre will be greatly lessened. It is nonsense to hold that health can only be maintained in the country. If towns were opened out enough they might still be healthy, even though they might be what are called "manufacturing towns" from end to end. We do not think that it is an ideal life for the bread-winner, whether as master or as man, to have to do his daily work in a crowded city far away from home, and practically a stranger to his family. Far better would it be that, by a spreading out of the towns themselves—their mills, their factories, their mansions, and their cottages—over a far larger area than is the case at present, a large majority of the people should live near their work. It is to be hoped that the example of Bristol will be largely followed. But if this is to be done, it is essential that suburban sanitary authorities should exercise far more care than they have hitherto done to maintain a proper sanitary standard, and to prevent the expansion of our great towns from taking the form of a disreputable fringe of narrow streets and crooked lanes, of pigsties, shanties, and slum dwellings—a fringe by which far too many of our great cities have in the past allowed themselves to be surrounded to the despair of the sanitarian of to-day.

### The Plague in Glasgow.

The appearance of plague in Glasgow is not calculated to occasion surprise in any who are acquainted with the facilities which exist for its importation, or with the state of the waterside population, among whom it would naturally tend to be first introduced; nor is it calculated to occasion alarm among any who have observed either the methods of dealing with imported disease which have prevailed in the United Kingdom for some years, or the results which these methods have afforded. Sir John Simon, a long time ago, in discussing the probable influence upon public health of the great modern developments of commerce and of facilities for locomotion, pointed out the reasonableness of expecting that the "contagions current in the East would soon become current in Europe"; and all subsequent experience indicates the absolute correctness of his forecast. There is no reason to doubt that plague, like cholera, will spread in any inhabited districts which are duly provided with the conditions necessary for the multiplication and diffusion of its bacillus, just as there is every reason to believe that it will not spread where the modes of life and the sanitary surroundings of the inhabitants make it difficult for the germs of the disease to obtain a footing. Thus one is brought back to the ever-standing question—why, in the midst of the general sanitary improvement so manifest throughout the country, districts should be left in which such dangerous conditions are allowed to prevail? There is a story of a Scottish minister, whose manse was in a deplorable sanitary state, such as to imperil his safety and that of his family, and who applied to the heritors of the parish (if that be the correct Scottish name for the territorial district) to ask that proper repairs and amendments might be undertaken without delay. Finding them unwilling to entertain the question, he obtained a report, in every

ANNOTATIONS—*continued.*

way confirmatory of his complaint, from an eminent sanitary authority; and, having sent this in, awaited with some impatience, but yet with complete confidence, what he regarded as the only possible result. In due time he was waited upon by a deputation of the heritors, who assured him of the attention which had been given both to his original complaint and to the convincing report by which it had been sustained, according to which the lives of the tenants of the manse were in danger; and wound up by saying, "We hae thoct it weel o'er, minister, and we hae come to the conclusion that we'll joost risk it." And it is to be feared that the sanitary authorities of Glasgow, notwithstanding the great improvements they have made, have yet been willing to "risk" something, in preference to insisting upon radical reforms in that portion of their waterside district which is chiefly inhabited by the poorest Irish. Now, however, all reasonable precautions are being carried into effect, and there seems good reason to hope, not only that the malady will be stayed, but also that the conditions which involve "risks" of its recurrence will be less mildly dealt with than they have been aforetime.

### The National Hospital for the Paralysed and Epileptic.

The storm at this hospital is by no means over, as is shown by the constant stream of letters which continue to be poured out by various persons interested in its welfare and good management, and we can hardly doubt that when the world returns to London, and finds time to deal practically with this important matter, some permanent and satisfactory arrangement will be arrived at. After the letter from Sir James Crichton-Browne, which appeared in the *Times* last Monday, it is obviously impossible that things can be left where they stand. He broadly asserts, in regard to the special general meeting held on August 11th, that, notwithstanding the protests of the medical staff, it was summoned "deliberately" for a date when it was impossible for any considerable proportion of the governors to attend, or for the medical staff to be represented; that its decision was taken on a statement of facts which Mr. G. W. E. Russell has since admitted to be incorrect, but which is still being circulated among the governors and subscribers by the authority of the board of management; that "it was a packed meeting, made up of friends of the board of management specially invited"; and that only 16 persons took part in the proceedings. It seems to us that there are three questions involved in the dispute—(1) Is the hospital well managed, so far as It seems to us that there are three questions involved in the dispute—(1) Is the hospital well managed, so far as regards its internal economy? (2) Ought the medical staff to be represented on the board of management? (3) Is it desirable that such an amount of control and power should be given into the hands of any one individual as are held to have fallen into the hands of Mr. Burford Rawlings? Now, as regards the first point, this is clearly not the main question, and if any deficiencies in the management of the hospital should be proved, they will be of interest chiefly as bearing upon the further question whether it is better that a hospital should be managed by a director or by a board. The real interest centres in questions (2) and (3), which involve problems in hospital management which are of importance not only to the National Hospital but to others, and it will be well that they should be thoroughly threshed out over this particular case. In the case of other hospitals the matter might be complicated by questions of capacity or position, but in this instance no one can doubt that the members of the medical staff are fully fitted for the highest seats on a hospital board if the principle of their admission be once admitted, while in regard to Mr. Burford Rawlings perhaps one may say that it is owing to his excellencies, and to the fact that he has shown himself so powerful an influence in the management of the National Hospital, that the questions seriously arise whether it is ever desirable to place in any one man's hands the powers attributed to him, and whether continuity of interest, and harmony between the different groups engaged in the conduct of a hospital, will not always be best secured by making a board on which all these interests are represented the one central source of power, without the intervention of any intermediary. These are questions which are of wide interest, quite apart from the particular hospital in regard to which they have arisen.

### Sir Redvers Buller on the Royal Army Medical Corps.

Some of the accusations which have been persistently made in regard to the work of the Royal Army Medical Corps have elicited from Sir Redvers Buller a letter to the *Times* last Monday, in which he bears striking testimony to the work done by the members of that corps. This letter is of special importance in that it deals with the point which is sure to be made sooner or later by those who have all along objected to the military titles and the military status now enjoyed by the medical officers. So far as the investigations have hitherto gone they have tended to throw the responsibility of any muddle that may have occurred upon the shoulders of the transport authorities, and upon those restrictive War Office rules which prevent medical officers "commandeering" the supplies they want. It has been demonstrated above all things that the doctors, orderlies, and nurses did not spare themselves, but, literally in many cases, worked themselves to death. What is called the "military clique," however, is sure to endeavour to fix upon the new rank and status of the doctors much of the blame of any failure which may be proved, and in regard to this point the words of Sir Redvers Buller are well worthy of note. He says: "Mr. Lees Knowles publishes and endorses, as the utterance of a person in high position, the following words: 'I am given to understand that the senior officers Royal Army Medical Corps, with few exceptions, are so taken up with their own importance as combatant officers, and their rank as colonels and majors, that they leave much to be desired.' Was ever a more shameful accusation more shamelessly published? There were then but three colonels Royal Army Medical Corps in Natal outside Ladysmith"—Colonel Gallwey, Colonel Clery, and Colonel Allin—and after detailing their services, he adds: "I could say as much for the next senior officers R.A.M.C., but colonels are mentioned, and I challenge Mr. Lees Knowles, and his correspondent of high position, to say which colonels they refer to, and either to prove their assertions or withdraw their slander." In Wednesday's *Times* Mr. Lees Knowles withdraws and expresses regret. The thanks of the service are due to Sir Redvers Buller for his vigorous adjectives.

